# White Blood Cell Enumeration and Differential by Flow Cytometry: The ICSH WBC Reference Method

**DOI:** 10.1111/ijlh.14553

**Published:** 2025-09-11

**Authors:** Benjamin D. Hedley, Michael Keeney, Peter Gambell, Chenxue Qu, Jenny Mao, Bruce H. Davis, Brent L. Wood

**Affiliations:** ^1^ London Health Science Center London Ontario Canada; ^2^ Peter McCallum Cancer Center Melbourne Australia; ^3^ Peking University First Hospital Beijing China; ^4^ University of Washington Seattle WA USA; ^5^ Trillium Diagnostics (Retired CEO) Bangor Maine USA; ^6^ Children's Hospital los Angeles Los Angeles California USA

**Keywords:** analyzer, flow cytometry, hematology, Immuno‐differential, reference

## Abstract

**Introduction:**

The current reference method for the white blood cell (WBC) differential is manual smear review as outlined in CLSI H20‐A2. As with many manual methods, it suffers from a number of challenges including dependence upon the expertise of the interpreter, the quality of the smear and stain, when dysplastic features make cell identification difficult, imprecision with leucopenia, and enumeration bias due to non‐uniform cell distribution.

**Methods:**

This study describes an alternative method for establishing the leucocyte differential using a single‐tube, 8‐color flow cytometric reference method.

**Results:**

Data presented is from an international comparison of normal (based on analyzer counts, *N* = 120) and abnormal (*N* = 496) clinical samples performed at four institutions using four different models of flow cytometers. Here we demonstrate equivalent performance between the flow cytometric method and the current manual reference method, but show improved performance of the proposed reference method for low/infrequent cell populations, for example, monocytes and basophils.

**Conclusion:**

The flow cytometric method also performs well in comparison with hematology analyzers in current clinical use, including good correlation for total white blood cell enumeration. The findings indicate that the flow cytometric method, deemed the “ICSH WBC reference,” could be used in lieu of CLSI H20‐A2 as a reference for white blood cell enumeration and differential counting and specifically for the evaluation of automated differential counters.

## 1 Introduction

The white blood cell (WBC) differential is an essential assay in the clinical laboratory that is used for screening and monitoring of a large number of clinical conditions ranging from infection to neoplasia. The primary method for generating the WBC differential is the automated hematology analyzer but is supplemented by morphologic or digital image‐based smear review. This is required in 10%–50% of samples due to uncertainties in cell identification as flagged by the hematology analyzer. Improvements in automated WBC differential counting require a robust reference method to accurately document and validate instrument performance, particularly when obtaining IVD regulatory approvals. The current reference method is manual smear review performed by two reviewers counting 200 cells each, as outlined in CLSI H20‐A2, first approved in 1992 and revised in 2007 [1] with a 2023 revision recently reviewed. The scope of CLSI H42 does include a flow cytometric method as a reference method; however, this application was limited to blood from healthy individuals. As with many manual methods, it suffers from a number of challenges. These include the level of training and experience of the interpreter, particularly when smear preparation and staining may be suboptimal, the presence of dysplastic features making cell identification difficult, imprecision with leucopenia, and non‐uniform cell distribution (Vis 2016) [2]. Advanced multi‐color flow cytometry allows for an alternative method that could potentially improve the manual reference method through evaluation of larger numbers of cells and more specific identification of cell types using antigenic identification.

A number of flow cytometric methods have been published demonstrating the feasibility of this approach for generating a WBC differential [3, 4, 5, 6, 7, 8, 9]. These methods show generally good concordance between the flow cytometric method and manual morphology and/or hematology analyzers for the standard cell types of a 5‐part differential provided by hematology analyzers, that is, neutrophils, lymphocytes, monocytes, eosinophils, and basophils. An initial comparison of three published flow cytometric candidate methods by an International Council for Standardization in Hematology (ICSH) Task Force demonstrated equivalent performance for these normal cell types and an extended differential assay including abnormal cell types [10]. Based on this experience and following a specification developed by the ICSH Board, a revised and updated assay was developed to also allow identification of immature granulocytes, blasts, nucleated red cells, and lymphocyte subpopulations (B cells, T cells and NK cells). This revised panel allows for specific identification of all lymphocytes. The resulting assay is here applied to a large number of normal (based on laboratory reference ranges) and patient‐derived clinical samples to demonstrate its accuracy and robustness in a multi‐institutional and international context when compared against both the manual morphologic reference method and standard hematology analyzers. This evaluation is intended as a real‐world comparison and so includes flow cytometers and hematology analyzers from multiple instrument manufacturers without any pre‐study cross‐validation or standardization between instruments.

The ultimate objective of the effort is to provide an alternative or potential replacement for CLSI H20‐A2 as a reference method for white blood cell enumeration and differential counting. The method is intended to be particularly useful for manufacturers of hematology analyzers as a reference method for instrument validation. It also has the potential to be used for validation of instruments and evaluation of problematic samples in the clinical laboratory. Noteworthy is that the intended use of this proposed reference method is not that of clinical practice, as it presents significant cost barriers.

## 2 Method

### 2.1 The Assay

The specification for the method provided by the ICSH Board advocated for the identification of the standard five populations currently identified by most hematology analyzers using a combination of positive and negative antigens: Lymphocytes, Monocytes, Granulocytes, Eosinophils, and Basophils. In addition, there was a desire to include Immature Granulocytes, Blasts (as defined morphologically), Nucleated Red Cells, and the Lymphocyte subpopulations including B cells, T cells, and NK cells. Lymphocyte subpopulations have no definitive morphologic features, but their identification was considered useful to confidently identify all lymphocytes. The assay described below meets these requirements.

**FIGURE 1:**
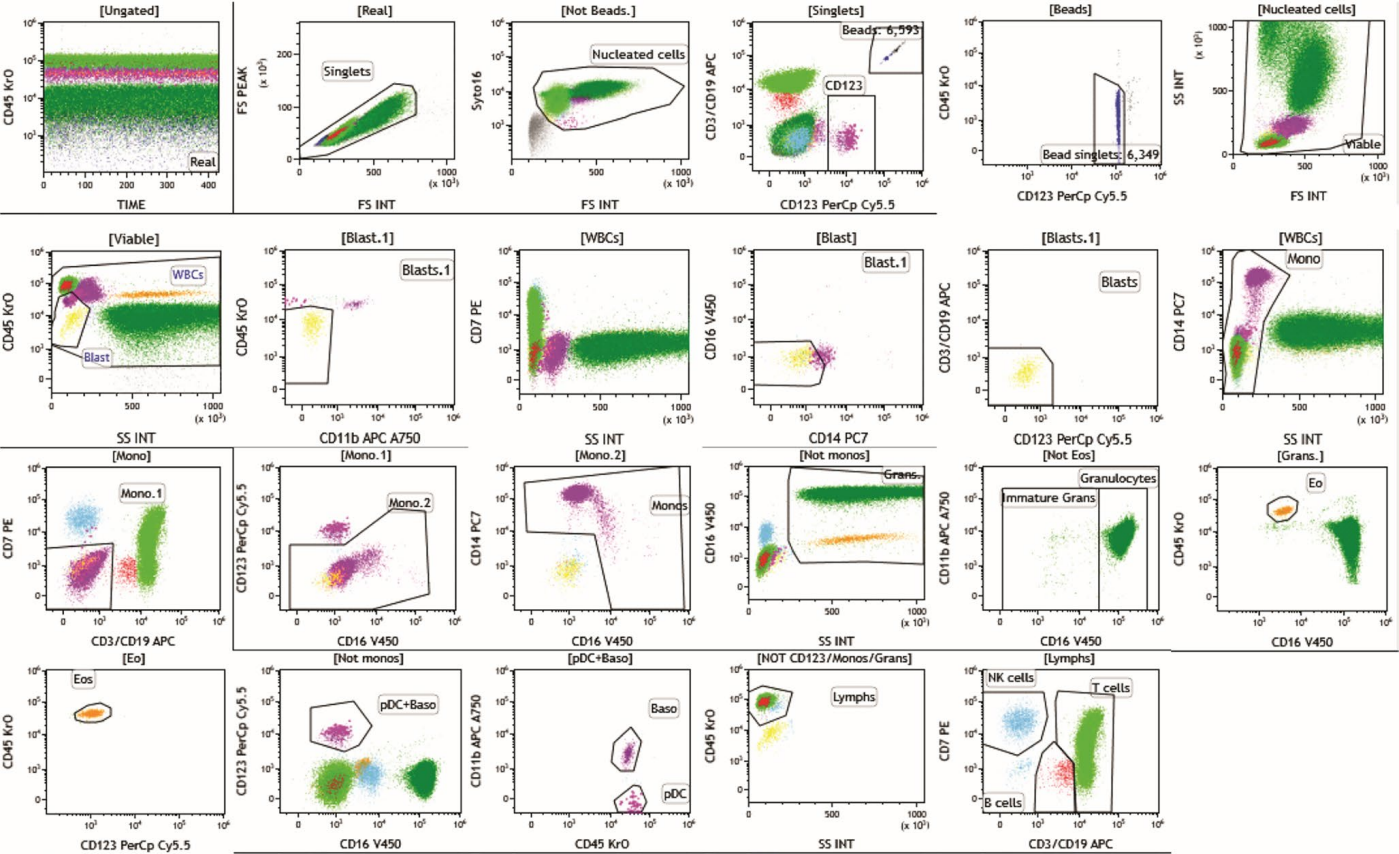
Flow cytometry data analysis example. An example of the sequential gating strategy used for this study is provided with the parent population label for each dot plot indicated above the dot plot corresponding to the labeled gates.

A detailed procedure is provided in the Supporting Information. Briefly, an antibody cocktail was added to a 100 μL aliquot of peripheral blood and processed using a Lyse/No‐Wash method in order to reduce cell loss and minimize sample manipulation. Absolute counting beads were included to provide direct enumeration of absolute counts for each population, particularly white blood cells, to allow direct comparison with a hematology analyzer. A permeant nucleic acid binding dye, Syto16 [11, 12], was included to definitively identify nucleated cells of all types. Following incubation, 100,000 nucleated (Syto16+) events were acquired on the cytometer at each institution. Six of the required cell populations were identified by positive expression of one or more antigens, with most cells identified by multiple antigens in a multiparametric gating strategy; see Table S1. One exception was blasts, which, due to their heterogeneous immunophenotypes in neoplastic conditions, were identified by exclusion. An example of the analysis strategy used to identify each of the populations is provided in Figure 1.

### 2.2 Assay Performance Characteristics

Assay sensitivity, linearity, and intra‐assay reproducibility were evaluated by performing serial dilution of three samples in triplicate. Inter‐assay reproducibility was evaluated by assay of a stabilized peripheral blood control material (Immunotrol, Beckman‐Coulter, Miami FL, USA) over 5 days. Specificity was evaluated as part of the multi‐institutional evaluation versus the CLSI H‐20 reference method as below. Sample stability was assessed by evaluating five samples at 0, 24, and 48 h. For all performance characteristics, a coefficient of variation of < 10% and *r* ^2^ values greater than 0.95 were deemed acceptable for the five major populations within peripheral blood.

### 2.3 Multi‐Institutional Evaluation of Accuracy and Robustness

Demonstration of the ability of multiple laboratories to successfully execute the assay and generate comparable data was a primary objective. Four laboratories were selected: University of Washington in Seattle, Washington, USA (Brent Wood), Peter MacCallum Cancer Center in Melbourne, Australia (Peter Gambell), London Health Sciences Center in London, Ontario, Canada (Michael Keeney and Benjamin Hedley), and Peking University First Hospital in Beijing, China (Chenxue Qu). The criteria for inclusion were international representation and the ability to successfully execute the assay and the study as a whole. Protection of human subjects was maintained according to national and international standards for the conduct of clinical studies, including 21CFR Parts 50 and 56 and International Conference on Harmonization (ICH) E6—Good Clinical Practice Consolidated Guideline. All data was acquired with de‐identified labels on the hematology and flow cytometry instruments. Each site received the same lots of reagents (e.g., antibodies, reagents, tubes) and titrations were determined at a single site (Seattle) and the resulting information distributed to all sites. Each institution submitted raw data on three samples for coordinator review (BW) prior to the collection of the accuracy dataset to ensure the assay was performing correctly. Each site was instructed to contribute 150 peripheral blood samples, including 30 patients with normal counts based on their reference ranges (normal was based on each institutional policy and guidance was given for cases to collect, see Supporting Information Materials). For each sample, the site provided the analyzed and raw flow cytometric assay results, hematology analyzer results (WBC count and WBC differential), and a manual WBC differential count performed according to the CLSI H20‐A2 (400 cells counted, 200 on each of two slides). To provide real‐world implementation experience, each site was provided the detailed methodology, including the gating strategy with an expectation to execute the procedure as written, but no attempt was made to cross‐standardize the laboratories. In aggregate, the instrumentation used between the sites included both Beckman‐Coulter (Navios EX) and Becton‐Dickinson (LSRII and FACSCanto) flow cytometers, and Beckman‐Coulter (DxH 800), Sysmex (XN) and Abbott (CellDyn Sapphire) hematology analyzers. Data for 616 samples was ultimately submitted and distributed roughly equally between the four laboratories. Samples were stained according to the protocol, and blood smears were made according to the guidance document CLSI H20‐A2 within 4 h of blood collection. Samples were excluded from the analysis if manual WBC differential could not be obtained; otherwise, samples were selected as normal based on the criteria listed above (*N* = 30) or abnormal based on hematology analyzer flagging. Importantly, slide classification was conducted according to each individual laboratory's practice; the only requirement was that two operators independently assess the slide and report the 200 cell differential according to the template provided. This template can be found as a table in Data S1.

### 2.4 Statistical Analysis

All sample subsets were analyzed for correlation coefficients and simple *R* ^2^ regression statistics. Biases (Y intercepts during regression analysis) of less than 1 × 10^3^ cells per microliter and *R* ^2^ value of greater than 0.90 were considered to be acceptable.

## 3 Results

The assay performance was found to be excellent, with sensitivity CVs being less than 10%, linearity *r* ^2^ values of over 0.95 for all populations (excepting basophils), and within‐run reproducibility showing variation of less than 5% for each of the five major populations (data not shown). Blast cells and immature granulocytes were also assessed for reproducibility and showed CVs of 16.1% and 20.3%, respectively, due in part to the low numbers seen in the samples. Assay stability was assessed at 24‐h increments over 2 days (three time points) and showed CVs all below the 10% outlined in the methods, indicating excellent performance over the 48‐h time period. Similarly, minor populations (in percentage, not with respect to clinical impact) were found to have higher CVs, with blast cells showing the largest value at just over 15% (data not shown).

**FIGURE 2:**
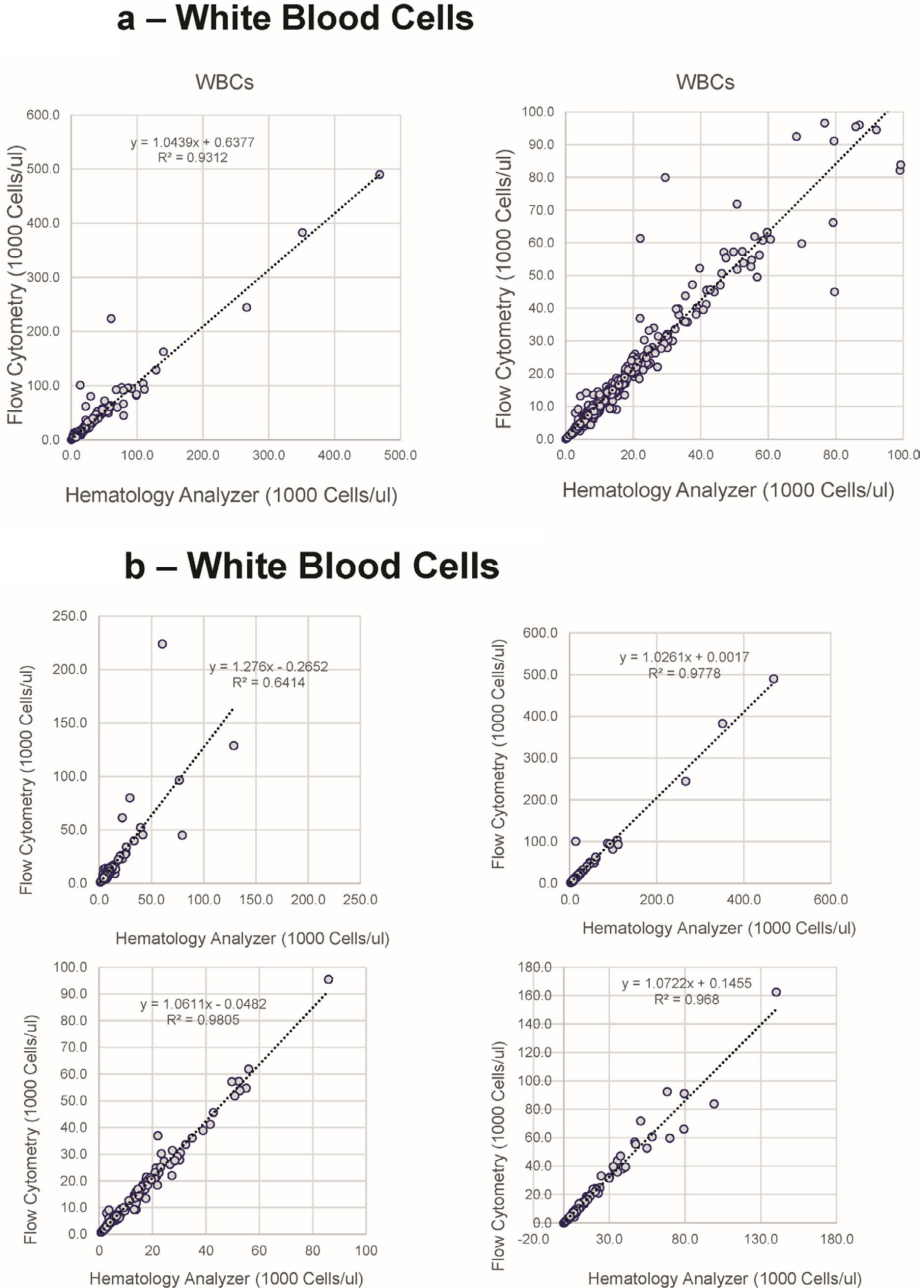
WBC count. (a) Correlation between the flow cytometric method and hematology analyzer overall the full range of samples (left) and those with WBC less than 100 000 cells/μL. (b) Correlation between the flow cytometric method and hematology analyzer for each of the four laboratories providing data.

The correlation between the WBC count provided by the flow cytometric assay and the hematology analyzers for all sites was excellent (Figure 2a,b) with a slope of 1.044 and *R* ^2^ of 0.9312. To assess the variability seen at high white counts for a small subset of samples, the data for each laboratory is separately provided (Figure 2b) and the variability appears derived from two of the laboratories, each contributing five deviant samples. Care should be taken when dilutions are made while performing dilutions, and for study purposes, large variations in results should be investigated, repeated if possible, and criteria for exclusion developed prior to the start of an instrument validation.

**FIGURE 3:**
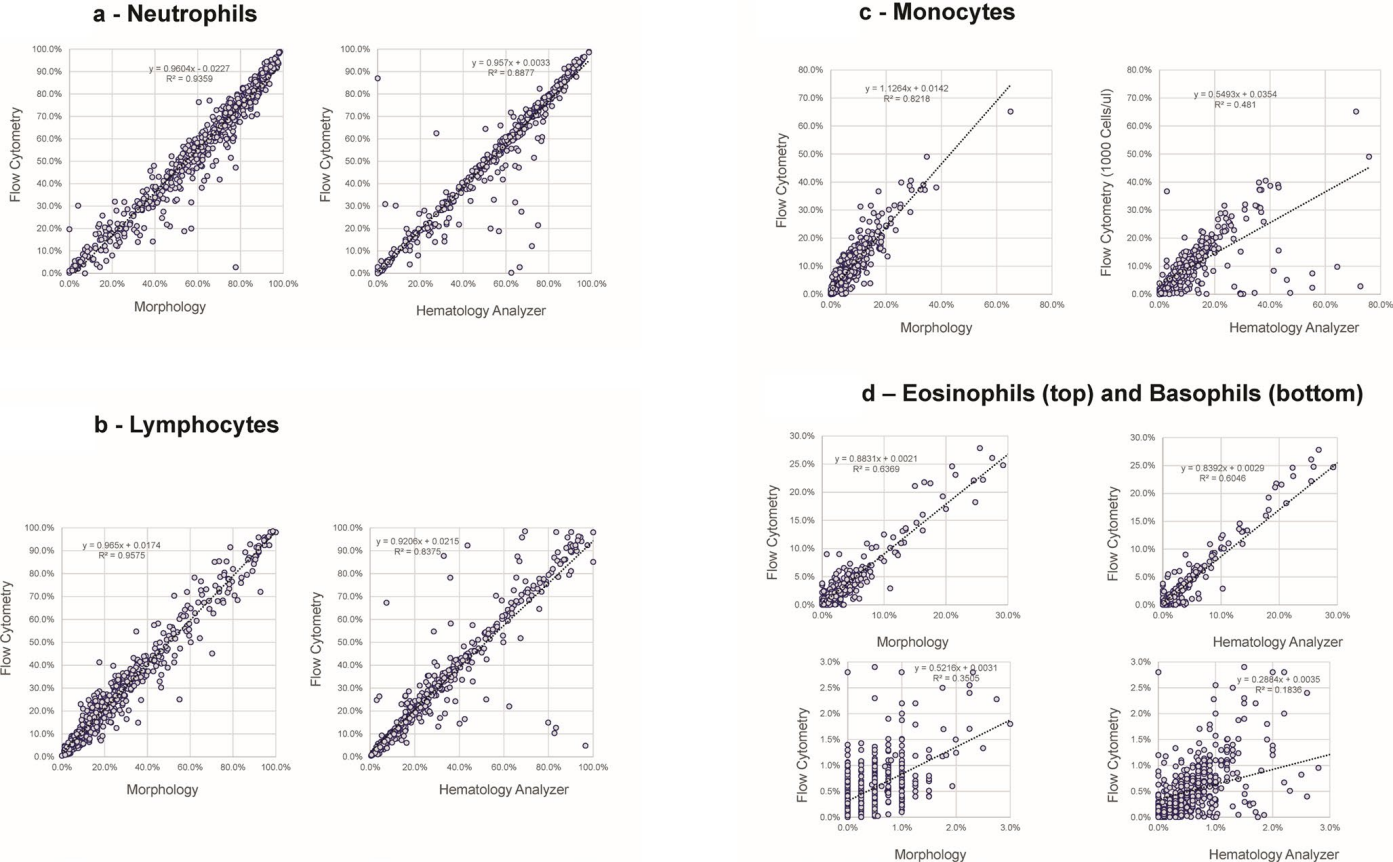
(a) Neutrophil percentage. Correlation between the flow cytometric method, morphology (left) and hematology analyzer (right). Note the increased values provided by the hematology analyzer for a subset of samples. (b) Lymphocyte percentage. Correlation between the flow cytometric method, morphology (left) and hematology analyzer (right). Note the increased values provided by the hematology analyzer for a subset of samples. (c) Eosinophil and Basophil percentages. Correlation between the flow cytometric method, morphology (left) and hematology analyzer (right) for eosinophils (top) and basophils (bottom) Note the generally poor correlation seen for basophils. (d) Eosinophil and Basophil percentages. Correlation between the flow cytometric method, morphology (left) and hematology analyzer (right) for eosinophils (top) and basophils (bottom) Note the generally poor correlation seen for basophils.

The correlation between the percentage of neutrophils provided by the flow cytometric assay and morphology for all sites shows generally excellent correlation with a slope of 0.960 and *R* ^2^ of 0.9359 (Figure 3a). A small subset of samples showed a relative increase in neutrophils for the hematology analyzers, compared with flow cytometry. As these outliers were less evident in the comparison between the morphologic differential and flow cytometry, the hematology analyzer was likely overestimating the neutrophil percentage for a subset of samples. A few samples showing slightly higher neutrophil percentages compared with flow cytometry were shown to be due to a slight inaccuracy in compensation settings (discovered when all the data was reviewed and corrected) on the flow cytometry, that prevented recognition of the entire neutrophil population, see Figure S1. The small number of samples affected by this did not significantly alter the correlation coefficient or the *R* ^2^ value for the neutrophils. In addition, a single sample shows marked deviation between the flow cytometry and both hematology analyzers and morphology due to a CD16 polymorphism, a well‐known but infrequent phenomenon [13].

The correlation between the percentage of lymphocytes provided by the flow cytometric assay and morphology for all sites showed excellent correlation with a slope of 0.965 and *R* ^2^ of 0.9575 (Figure 3b). A small subset of samples showed a relative increase in lymphocytes for the hematology analyzers compared with flow cytometry. As these outliers were less evident in the comparison between the morphologic differential and flow cytometry, the hematology analyzer may again have been overestimating the lymphocyte percentage for a subset of samples. The absolute lymphocyte counts also show good correlation between the hematology analyzers and flow cytometers with only seven outliers above 20,000 cells/μL (a value that is outside the normal parameters) that are largely higher on the hematology analyzers (data not shown).

The correlation between the percentage of monocytes provided by the flow cytometric assay and morphology for all sites (Figure 3c) showed a very good correlation with a slope of 1.1264 and *R* ^2^ of 0.8218. There were roughly 15 samples showing a relative increase in monocytes for the hematology analyzers compared with flow cytometry, likely caused by overestimation of the monocyte percentage for a subset of samples on the hematology analyzer. The absolute monocyte counts showed poor correlation between the hematology analyzers and flow cytometers due to the same outliers, which have absolute monocyte counts above 10,000 cells/μL that are significantly higher on the hematology analyzers (data not shown).

The correlation between the percentage of eosinophils provided by the flow cytometric assay and morphology for all sites (Figure 3d) showed very good correlation with a slope of 0.8831 and *R* ^2^ of 0.6369. The absolute eosinophil counts show very good correlation between the hematology analyzers and flow cytometers (data not shown). However, the correlation between the percentage of basophils provided by the flow cytometric assay and morphology for all sites is provided in Figure 3d and shows very poor correlation, in part due to the relatively small number of basophils counted by morphology and higher specificity provided by flow cytometry for this population. Similar poor correlation for basophil percentage and basophil absolute count is seen between the flow cytometric assay and the hematology analyzers, likely largely due to poor specificity for basophils on the part of the hematology analyzers, a known issue, as well as the low number of specimens with high basophil counts.

**FIGURE 4:**
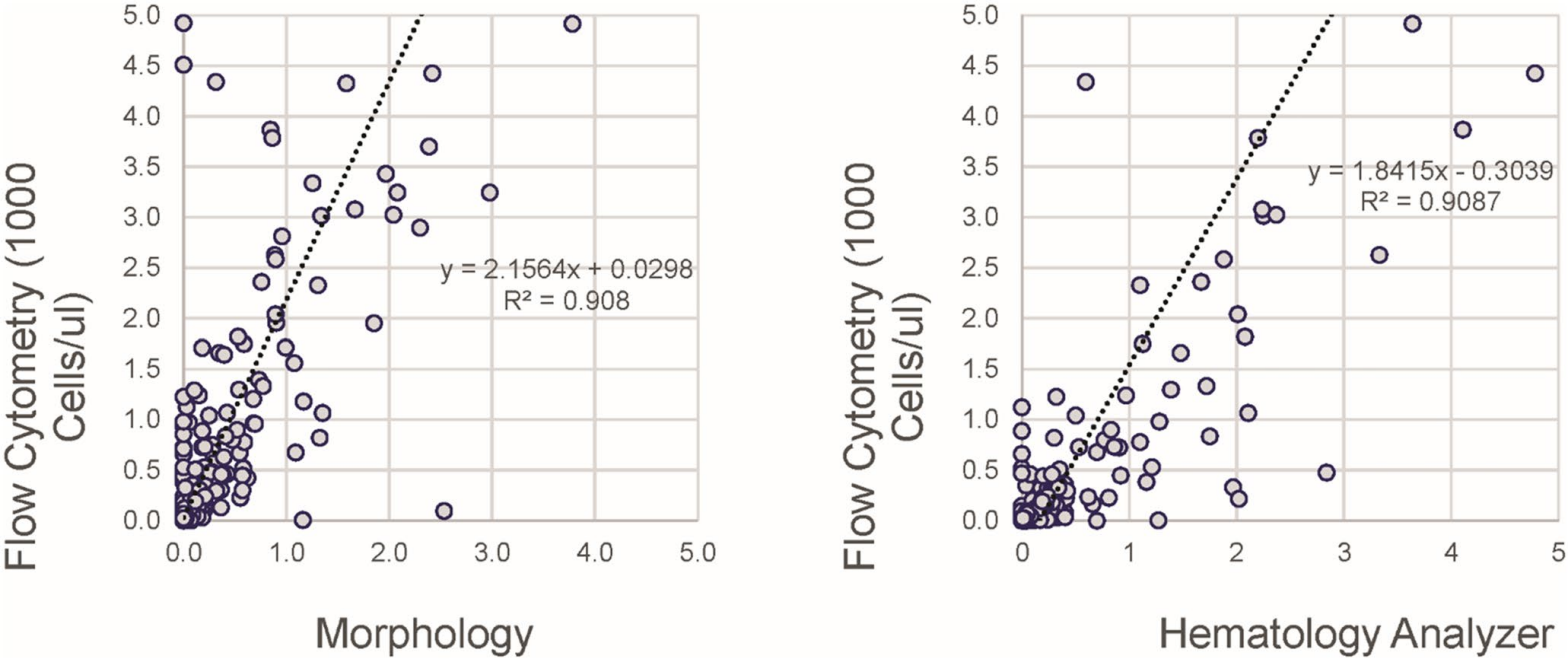
Immature granulocyte percentage. Correlation between the flow cytometric method, morphology (left) and hematology analyzer (right). Note the increased values provided by the hematology analyzer for a subset of samples (right).

The correlation between the absolute counts of immature granulocytes provided by the flow cytometric assay and morphology for all sites showed only fair correlation with a slope of 2.1564 and *R* ^2^ of 0.908 (Figure 4). The variability is in part due to the relatively small number of immature granulocytes counted by morphology and possibly the high variation between morphologists in differentiating band and segmented neutrophils, but also due to reduced discrimination between mature and flow cytometric immature granulocytes due to some variability in CD16 expression on mature granulocytes used for thresholding (Figure S2). Many samples showed a relative increase in immature granulocytes for the hematology analyzers compared with flow cytometry, probably due to overestimation by the hematology analyzers, which rely on less specific characteristics, largely light scatter. Absolute immature granulocyte counts show marginally improved correlation between all methods, although somewhat better correlation between the flow cytometric and morphologic methods was observed (data not shown).

**FIGURE 5:**
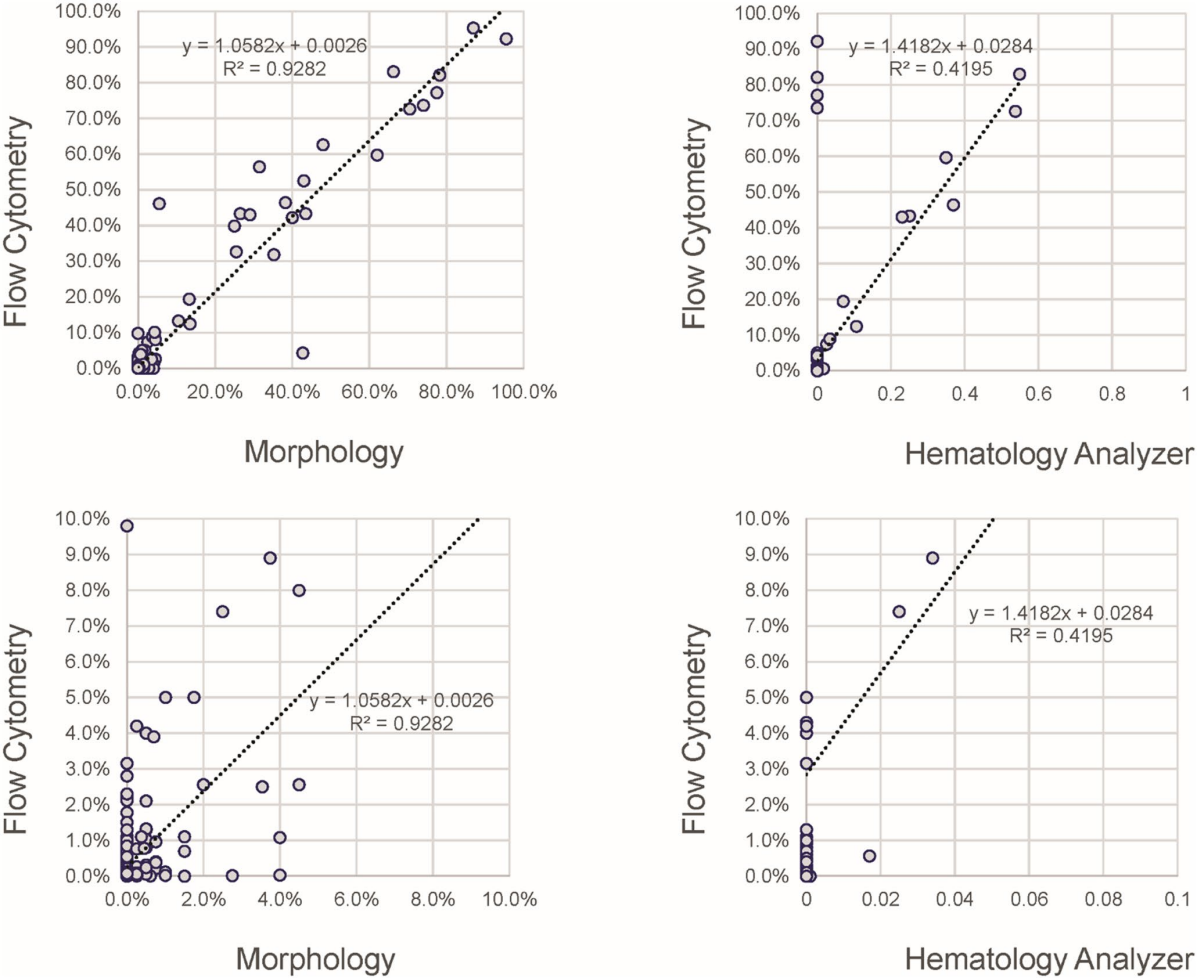
Blast percentage. Correlation between the flow cytometric method, morphology (left) and hematology analyzer (right) over the full range of sample (top) and those with less than 10% blasts (bottom). Note the generally poor correlation for samples with less than 10% blasts.

Blasts are interpreted by instrumentation as unclassifiable cells and alert operators through flagging and operator alerts, making a numerical correlation between an analyzer and any other method impossible. The correlation between the percentage of blasts provided by the flow cytometric assay and morphology for all sites is provided in Figure 5 and shows good correlation above 10% with an overall slope of 1.0582 and *R* ^2^ of 0.9282. Below 10% blasts or a blast count of 1.0 cells/μL the correlation was poor, with a high degree of variability by both assays, in part due to the relatively lower number of blasts counted and the use of exclusion to identify blasts by flow cytometry. The hematology analyzers showed poor correlation with flow cytometry for blasts, with a subset of samples having high blast percentages by flow cytometry and morphology reported as having no blasts by the hematology analyzer. Absolute blast counts show reasonably good correlation between flow cytometry and morphology above an absolute blast count of 1.0 cells/uL, but poor correlation below that threshold, similarly observed with the hematology analyzers.

## 4 Discussion

The assay described in this study is a single, lyse no‐wash, eight‐color tube and includes the nucleic acid binding dye Syto‐16 with the addition of enumeration beads to provide absolute counts on all gated populations in peripheral blood. The markers within this panel can enumerate six cell populations using positive markers and progenitors or blasts defined by exclusion. Nucleated red blood cells, which may be present under certain pathologies in peripheral blood, can be readily identified by the absence of CD45 and other antigens, positivity for Syto16, and low forward scatter, but were not directly evaluated in this study. Overall, the correlation between the flow cytometric assay and the morphologic reference method was very good for mature cell types in the peripheral blood with the exception of basophils where the flow cytometric method was judged to be superior due in part to its counting of larger numbers of cells. The correlation between the flow cytometric assay and the hematology analyzers was also very good for mature cell types except for basophils where the flow cytometric method is again judged to be superior due to the use of characteristics that allow more specific cell identification. Immature granulocytes showed greatest variability between all three methods, likely due to a combination of low population frequency and definitional issues in separating mature from immature granulocytes for each method (neutrophils/bands vs. metamyelocytes for morphology, use of CD16 for flow cytometry, use of light scatter for hematology analyzers) and the lack of a traceable standard for what are arbitrary morphologic features. Differences between automated and manual methods for identifying immature myeloid cells have been noted by others, likely for similar reasons [14]. Blasts or progenitors correlated well by both morphology and flow cytometry when large populations were present (> 10% or > 1 cell/uL), but below this level suffer from the large degree of imprecision primarily with morphology due to the small number of cells counted. The reduced specificity for flow cytometric blast or progenitor identification using exclusion is somewhat offset by improved precision given the much larger number of cells evaluated and likely provides somewhat more accurate enumeration below 10%. Similar findings were seen for blast enumeration using the CytoDiff method [15]. Hematology analyzers are confirmed to perform poorly for blast or progenitor identification through operator alerts (without any enumeration) likely due to the use of less specific cell characteristics that strongly overlap with more mature cell populations.

Patient samples are known to occasionally contain cell populations that may not be reliably detected by this assay. The design of this assay excluded a few cell types that are less frequently seen in peripheral blood, for example, megakaryocytes, mast cells, and plasma cells, in contrast to the positive identification of plasma cells in the methods of Bjornsson and Van de Geijn [6, 7]. There are also disease states or known infrequent variants in the population where the assay would be expected to have difficulty, examples being paroxysmal nocturnal hemoglobinuria due to loss of GPI‐linked proteins (CD14, CD16), CD16 polymorphisms on neutrophils (one example seen in the present study), plasma cell neoplasms, and mast cell neoplasms. Additionally, disease states that cause large numbers of nucleated red blood cells to be present in the peripheral circulation would be expected to be correctly identified by this method, but such samples were not seen in the current dataset and were not evaluated by the present study.

The assay presented in this study compares favorably to other published assays for this purpose [3, 4, 5, 6, 7, 8, 9, 16]. It provides a comprehensive approach in allowing determination of absolute counts for white cells and their subpopulations, definitive identification of all nucleated cells through use of a nucleic acid binding dye, and positive identification of the five main white cell subpopulations currently provided by hematology analyzers in addition to immature granulocytes, nucleated red cells, and blasts. This method has similarities to the proposed method from the Japanese Society for Laboratory Hematology [9]. The JSLH method is a published and reproducible method specialized for assigning reference values to fresh blood reference material (calibrators) from healthy individuals, although a nucleic acid binding dye is not included for nucleated cell identification and immature cell populations such as immature myeloid cells and blasts are not evaluated. Nevertheless, that method performed well in a multi‐institutional evaluation for imprecision and accuracy on a modest number of normal samples; its performance on abnormal samples commonly encountered in tertiary care environments remains to be evaluated. The JSLH method has also recently been used as part of a multimodal evaluation of a new hematology analyzer, although only limited data was presented for the flow cytometric method [2]. While it is likely that other published methods may perform similarly, the major strength of the current method is its evaluation on a large number of both normal and abnormal samples in a multi‐institutional and international context in comparison to both the current manual reference method and multiple hematology analyzers in common clinical use. As such, this represents a confirmation of the method's performance and robustness on many challenging clinical samples regardless of variations in instrumentation and laboratories.

The goal in assessing this flow cytometric method was to demonstrate equivalence to the current morphologic manual differential reference method outlined in CLSI H20‐A2. The data presented strongly support this conclusion and suggest that this flow cytometric method could supplant morphology as the reference method for white cell differential enumeration. Hematology analyzers have improved significantly over the past two decades and now offer rapid screening of samples and the ability to eliminate a large percentage of samples for further analysis. The use of a flow cytometric method having less subjectivity and a higher likelihood for automation provides hematology instrument manufacturers a more robust and objective standard against which to efficiently evaluate new analyzers, new algorithms, and improve on their ability to identify those samples that require more investigation. The application of this assay to instrument validation within a single laboratory setting was recently demonstrated [17]; this study further documents its robust and accurate performance characteristics in a multi‐institutional and international context. This method could also be used in the routine hematology laboratory for hematology analyzer validation at the time of installation and, with further refinement, could potentially be used to clarify difficult samples identified by either instrument flagging or morphologic slide review. It could also be used for more accurate value assignments for proficiency testing material and allow for accuracy‐based inter‐laboratory evaluations as opposed to the current peer group EQUA schemes.

## Supporting information


**Figure S1:** Outlier analysis for percentage neutrophils. A subset of outliers were investigated as to the cause for discordance between morphology and flow cytometry. Two points were discordant due to inaccurate compensation settings between v450 and KrO causing a subset of neutrophils to not be captured in the gate (top right). One sample showed a CD16 polymorphism that prevented recognition as neutrophils by flow cytometry (bottom right). One sample showed a likely overestimation of neutrophils by flow cytometry in a markedly abnormal sample that would require more careful gating for accuracy.

**Figure S2:** Gating of immature neutrophils. Most samples show a well‐defined neutrophil population with uniform CD16 expression that is easily gated (top left). A subset of samples show more variable CD16 expression reflecting maturational state whose threshold for discrimination between mature and immature forms more clear is some samples (top right) than others (bottom).

**Table S1:** Reagents and specificities used in the flow cytometric assay.

**Data S1:** supporting Information.

## Data Availability

The data that support the findings of this study are available from the corresponding author upon reasonable request.
