# Supplementary figures and images for: White Blood Cell Enumeration and Differential by Flow Cytometry: The ICSH WBC Reference Method
Source: Int J Lab Hematol. 2025 Sep 11;48(1):93–101. doi: 10.1111/ijlh.14553 (PMC12809377; doi:10.1111/ijlh.14553)

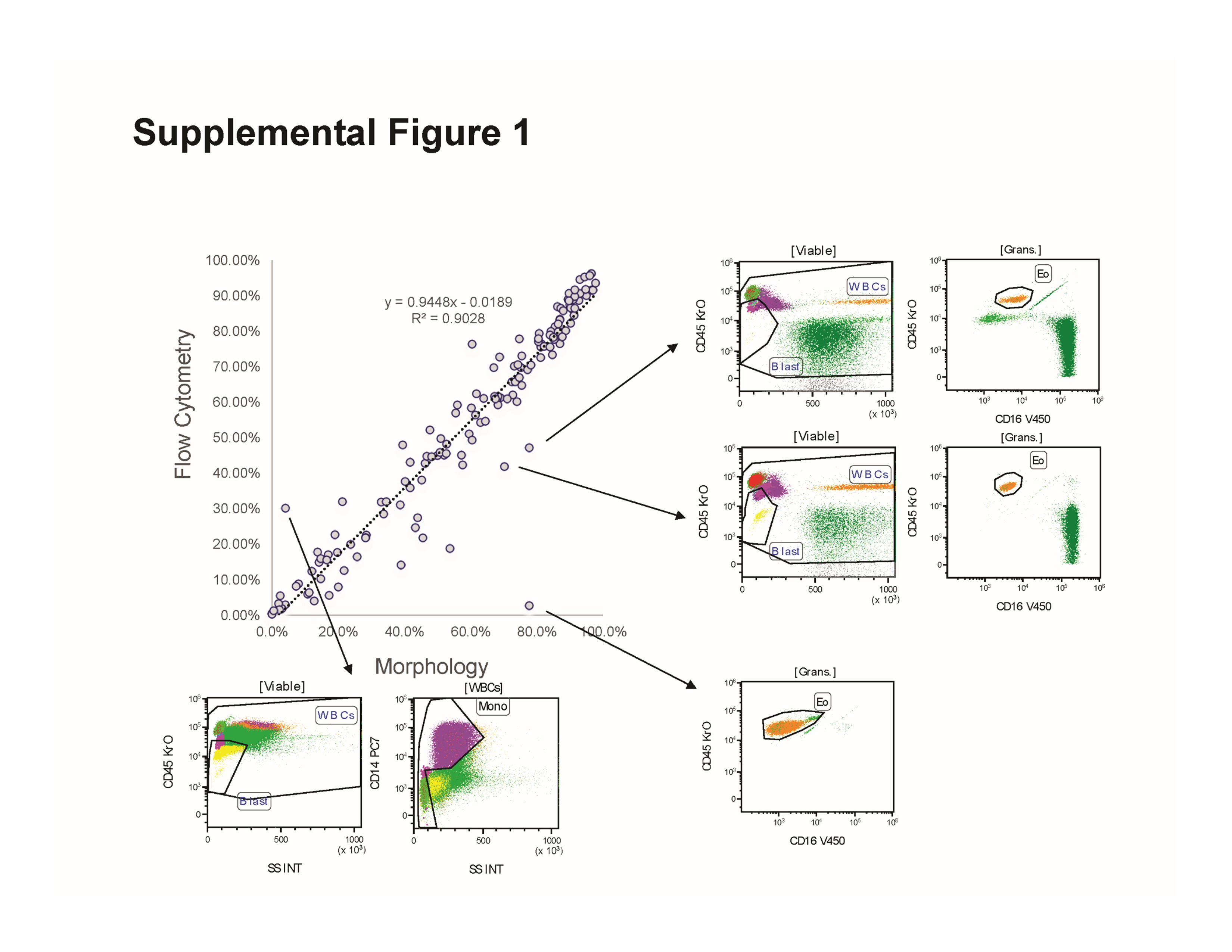

Supplement: Supplementary file 1 — Figure S1: Outlier analysis for percentage neutrophils. A subset of outliers were investigated as to the cause for discordance between morphology and flow cytometry. Two points were discordant due to inaccurate compensation settings between v450 and KrO causing a subset of neutrophils to not be captured in the gate (top right). One sample showed a CD16 polymorphism that prevented recognition as neutrophils by flow cytometry (bottom right). One sample showed a likely overestimation of neutrophils by flow cytometry in a markedly abnormal sample that would require more careful gating for accuracy. [file IJLH-48-93-s002.tif]

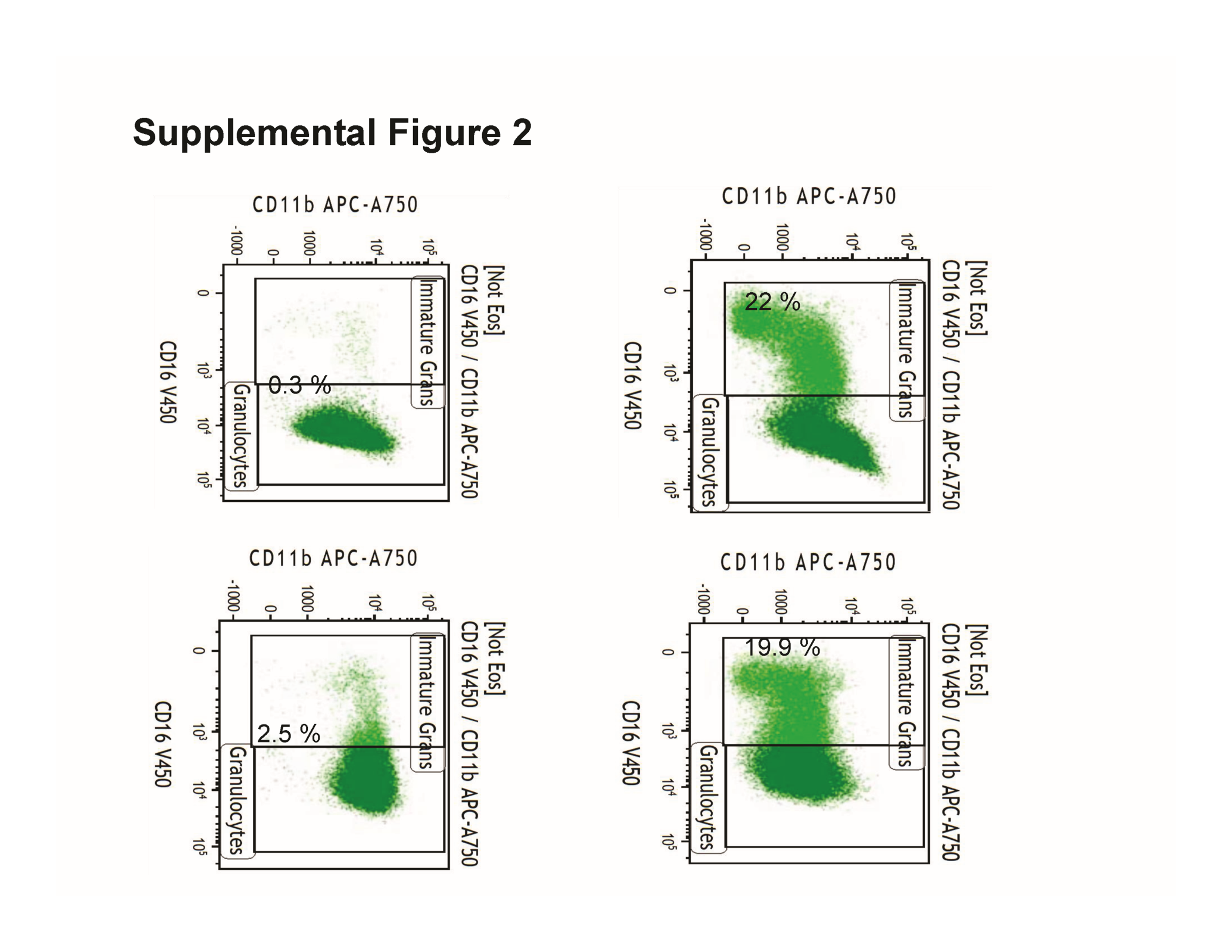

Supplement: Supplementary file 2 — Figure S2: Gating of immature neutrophils. Most samples show a well‐defined neutrophil population with uniform CD16 expression that is easily gated (top left). A subset of samples show more variable CD16 expression reflecting maturational state whose threshold for discrimination between mature and immature forms more clear is some samples (top right) than others (bottom). [file IJLH-48-93-s003.tif]
