# Supplementary material for: White Blood Cell Enumeration and Differential by Flow Cytometry: The ICSH WBC Reference Method
Source: Int J Lab Hematol. 2025 Sep 11;48(1):93–101. doi: 10.1111/ijlh.14553 (PMC12809377; doi:10.1111/ijlh.14553)
Supplement: Supplementary file 3 — Table S1: Reagents and specificities used in the flow cytometric assay. [file IJLH-48-93-s004.docx]

**Supplemental Table**

Table S1. Reagents and specificities used in the flow cytometric assay.

| **Reactivity** | **Fluorochrome** | **Population identified** |
| --- | --- | --- |
| Nucleic Acid | Syto16 | Nucleated cells |
| CD45 | Krome Orange (KrO) | Leukocytes |
| CD3 | Allophycocyanin (APC) | Lymphocytes – T Cells |
| CD19 | APC | Lymphocytes – B cells |
| CD7 | Phycoerythrin (PE) | Lymphocytes – NK and T cells |
| CD123 | Peridinin chlorophyll protein – Cyanine 5.5  (PerCp-Cy5.5) | Basophils, plasmacytoid dendritic cells |
| CD14 | PE-Cyanine 7 (PE-Cy7) | Mature monocytes |
| CD16 | V450 | Neutrophils vs. immature myeloid cells, NK cells, non-classical monocytes |
| CD11b | APC-A750 | Myeloid cells, monocytes, basophils, NK cells |
