# Supplementary material for: White Blood Cell Enumeration and Differential by Flow Cytometry: The ICSH WBC Reference Method
Source: Int J Lab Hematol. 2025 Sep 11;48(1):93–101. doi: 10.1111/ijlh.14553 (PMC12809377; doi:10.1111/ijlh.14553)
Supplement: Supplementary file 4 — Data S1: supporting Information. [file IJLH-48-93-s001.docx]

**Supplemental Materials**

1. Assay Procedure.

Assay Reagents

1. Monoclonal Antibodies

| **Specificity** | **Fluorochrome** | **Antibody Clone** | **Manufacturer**  **Product No. *** | **20 Test Cocktail (μl)** |
| --- | --- | --- | --- | --- |
| CD16 | V450 or PB | 3G8 | BD 644490  BD 558122 | 50 |
| CD45 | KO | J.33 | BC A96416 | 100 |
| CD7 | PE | M-T701 | BD 340656 | 200 |
| CD123 | PerCP-Cy5.5 | 7G3 | BD 558714 | 100 |
| CD14 | PE-Cy7 | RMO52 | BC A22331 | 20 |
| CD3 | APC | SK7 | BD 340661 | 50 |
| CD19 | APC | SJ25C1 | BD 340722 | 50 |
| CD11b | APC-A750 | BEAR1 | BC A97052 | 100 |
|  |  |  |  | Total:670  (33.5 **μ**l per test) |

* Manufacturer and catalogue numbers are US. BD=Becton Dickinson, BC=Beckman Coulter.

1. Versalyse (Beckman-Coulter IM3648).

Use as per instructions for use.

C. 0.25% Formaldehyde Preparation (PolySciences 04018).

Add 375 **μ**L of 10X Ultrapure Formaldehyde and QS to 15mL with distilled water. Make Fresh Daily.

D. Syto16 (Invitrogen S7578)

Prepare an initial dilution of 1:50 using 10 **μ**L of stock SYTO16 and 490 **μ**L of DMSO to achieve a concentration of 20 **μ**M (this can then be frozen for later use).

Add 10 **μ**L of 20 **μ**M stock to 190 **μ**L of PBS to make the working dilution.

E. BD Trucount Tubes (BD 340334)

Assay Procedure

1. Reverse pipette 100 **μ**L of peripheral blood into a BD Trucount Tube.

Note: Bood with a total leukocyte count of greater than 1 x103/μl dilute with 5% BSA

1. Add monoclonal antibodies at titer indicated in Table A. (*Note: alternatively add 33.5 **μ**L of prepared cocktail.)
2. Vortex vigorously for 3 seconds.
3. Incubate for 15 minutes at room temperature in the dark.
4. Add 1 mL of Versalyse and 400 **μ**L of 0.25% Formaldehyde.
5. Vortex for 3 seconds, being careful to not splash.
6. Incubate for 15 minutes at room temperature in the dark.
7. Add 5 **μ**L of Syto16 if WBC count is at or above 1.0x10^3^/**μ**L, add 2.5 **μ**L of Syto-16 if WBC count is below 1.0x10^3^/uL.
8. Vortex for 3 seconds, being careful to not splash.
9. Incubate for 15 minutes at room temperature in the dark.
10. Vortex to resuspend the beads prior to acquisition.
11. Run on Flow Cytometer and collect 100,000 Syto-16 positive events by gated acquisition using display of FS vs Syto-16.
12. Export data and analyze.

Variations:

1. BC FlowCount fluorospheres (BC product number 6607007) may be used in place of BD TruCount tubes, but requires additional reverse pipetting of the bead solution.
2. BD FACSlyse or NH_4_Cl containing 0.25% Formaldehyde may be used in place of Versalyse.
3. Other antibody conjugates having the same specificity and fluorochrome may be equally suitable, but require independent validation.
